# Removal of Aflatoxins Using Agro-Waste-Based Materials and Current Characterization Techniques Used for Biosorption Assessment

**DOI:** 10.3389/fvets.2022.897302

**Published:** 2022-05-16

**Authors:** Alma Vázquez-Durán, María de Jesús Nava-Ramírez, Guillermo Téllez-Isaías, Abraham Méndez-Albores

**Affiliations:** ^1^Unidad de Investigación Multidisciplinaria L14 (Alimentos, Micotoxinas, y Micotoxicosis), Facultad de Estudios Superiores Cuautitlán, Universidad Nacional Autónoma de México, Mexico City, Mexico; ^2^Department of Poultry Science, University of Arkansas, Fayetteville, AR, United States

**Keywords:** aflatoxins, agro-waste-based materials, biosorption, characterization techniques, decontamination

## Abstract

Aflatoxins are the most hazardous fungal-generated secondary metabolites produced by toxigenic *Aspergillus* species. These toxins are frequently detected in food and feed and impose either acute or chronic effects in humans and animals, causing great public concern. Because of the adverse effects of aflatoxins, many physical, chemical, and biological decontamination approaches have been developed. However, the most commonly used procedure is the addition of adsorbent materials into aflatoxin-contaminated diets to reduce toxin absorption and distribution to blood and target organs. In recent times, sorption technology with agro-waste-based materials has appeared as a promising alternative over conventional binding agents with the benefits of low cost, higher rentability, feasibility, and exceptional efficiencies. This review is mainly focused on discussing the most important agro-waste-based materials able to adsorb aflatoxins such as pomaces, seeds, stems, hulls, peels, leaves, berries, lignins, fibers, weeds, and various horticultural byproducts. Further data of the *in vitro, in vivo*, and *in silico* efficacy of these biomaterials to adsorb and then desorb aflatoxins are given. Besides, an overview of the main characterization techniques used to elucidate the most important physical and chemical mechanisms involved in the biosorption is presented. Finally, conclusions and future research necessities are also outlined.

## Introduction

Mycotoxins are a group of low molecular weight substances synthesized during the secondary metabolism of toxigenic fungi. These metabolites vary from simple compounds like moniliformin to very complex chemical structures such as the macrocyclic hexapeptide mycotoxins (1). Up to now, approximately 400 mycotoxins are known (2); however, scientific attention is mainly focused on those of greatest public health and agro-economic importance, such as aflatoxins, ochratoxins, patulin, fumonisins, trichothecenes (nivalenol, deoxynivalenol, T-2 and HT-2 toxins), and zearalenone. These mycotoxins account for millions of dollars in annual losses because these compounds may exert severe adverse health effects in both humans and animals.

**Figure 1:**
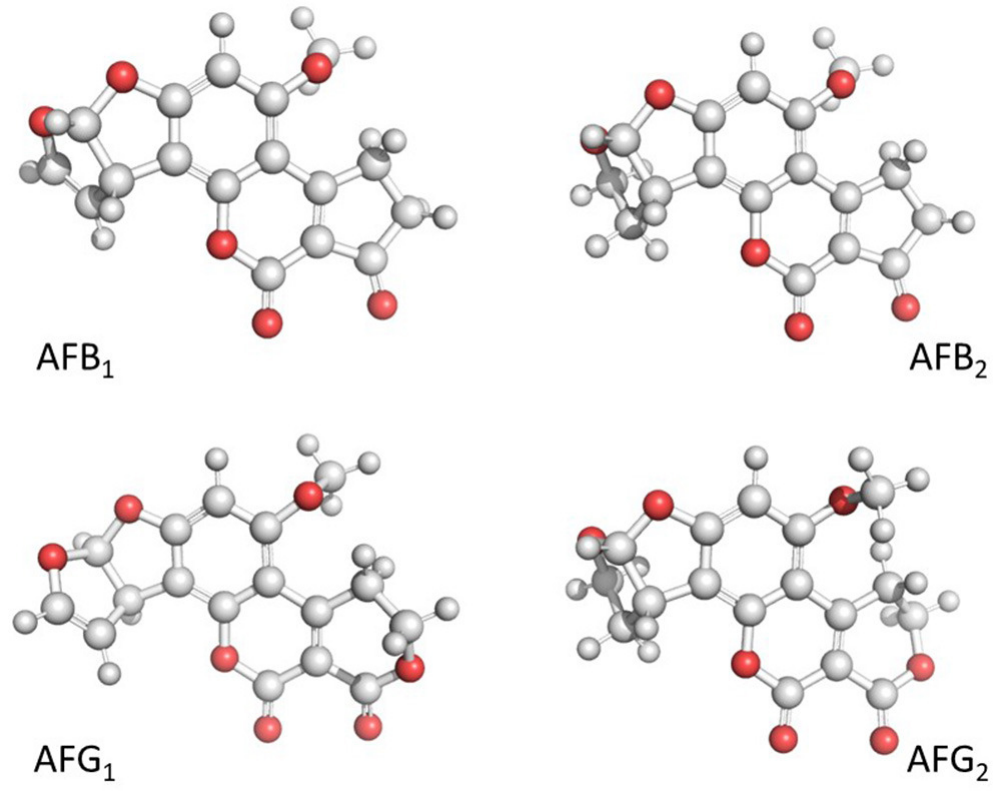
3D visualization of the chemical structure of aflatoxin B_1_ (AFB_1_), aflatoxin B_2_ (AFB_2_), aflatoxin G_1_ (AFG_1_), and aflatoxin G_2_ (AFG_2_). Created with BioRender.com.

Aflatoxins are furanocoumarin derivatives produced by several species of *Aspergillus* section *Flavi* (3). Four principal aflatoxins are produced (Figure 1); *Aspergillus togoensis* synthesize aflatoxin B_1_ (AFB_1_) only; *A. flavus* and *A. pseudotamarii* synthesize aflatoxin B_1_ (AFB_1_) and aflatoxin B_2_ (AFB_2_); while *A. aflatoxiformans, A. arachidicola, A. austwickii, A. cerealis, A. luteovirescens, A. minisclerotigenes, A. mottae, A. nomius, A. novoparasiticus, A. parasiticus, A. pipericola, A. pseudocaelatus, A. pseudonomius, A. sergii*, and *A. transmontanensis* produce AFB_1_, AFB_2_, aflatoxin G_1_ (AFG_1_), and aflatoxin G_2_ (AFG_2_) (4). AFB_1_ has a range of biological activities such as acute toxicity, teratogenicity, mutagenicity, and carcinogenicity (5); consequently, aflatoxins, including AFB_1_, AFB_2_, AFG_1_, AFG_2_, and aflatoxin M_1_ (AFM_1_) have been classified as carcinogenic to humans (Group 1) by the International Agency for Research on Cancer (6). As many excellent reviews on aflatoxins already exist in the literature that outline the biosynthesis, ecology, metabolism, chemical structure, biological effects, toxicity, occurrence, detection, control, detoxification, and legislation, only these few lines will be presented herein.

**Figure 2:**
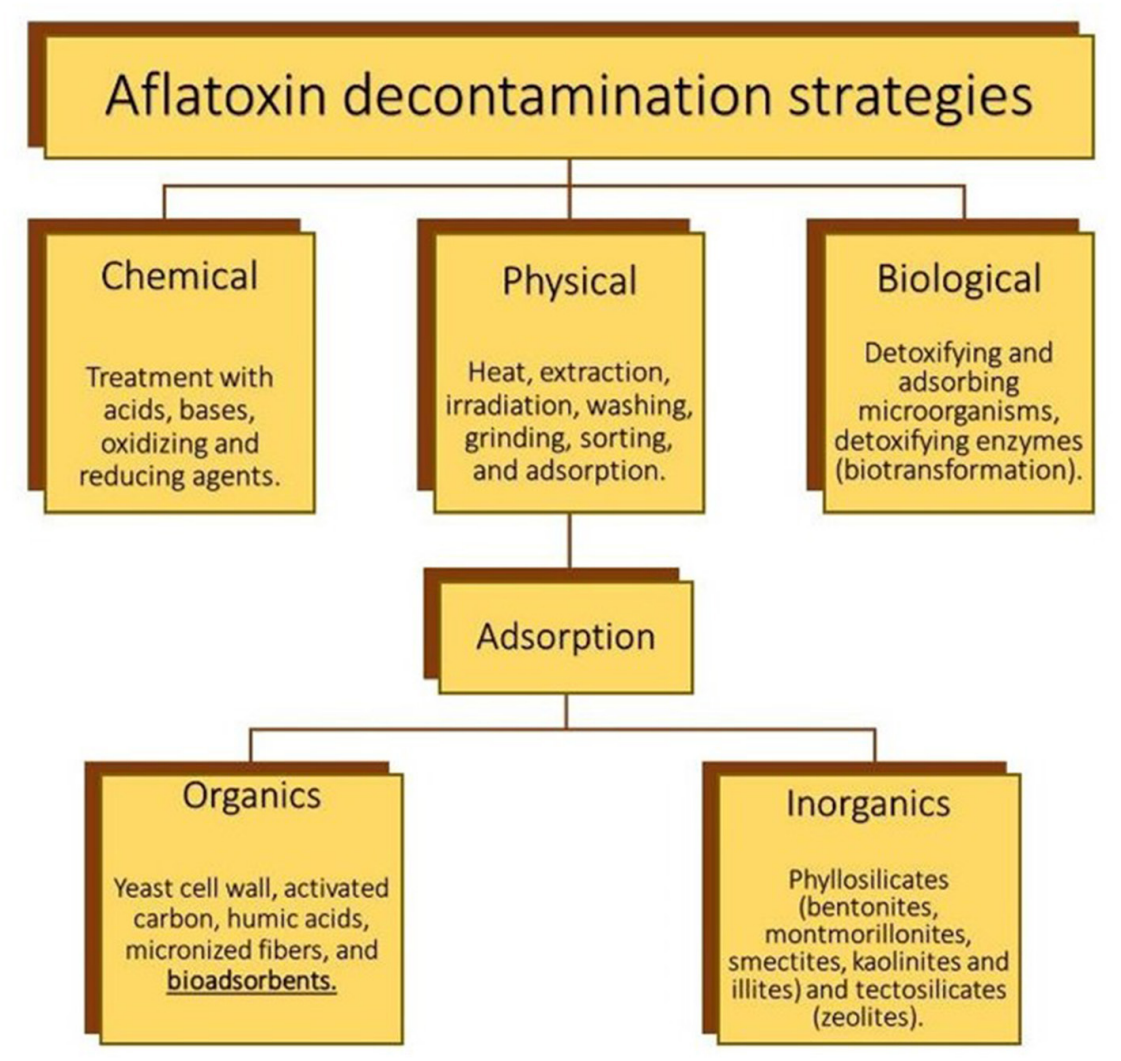
The most important physical, chemical, and biological decontamination technologies for aflatoxin control. Created with BioRender.com.

**Figure 3:**
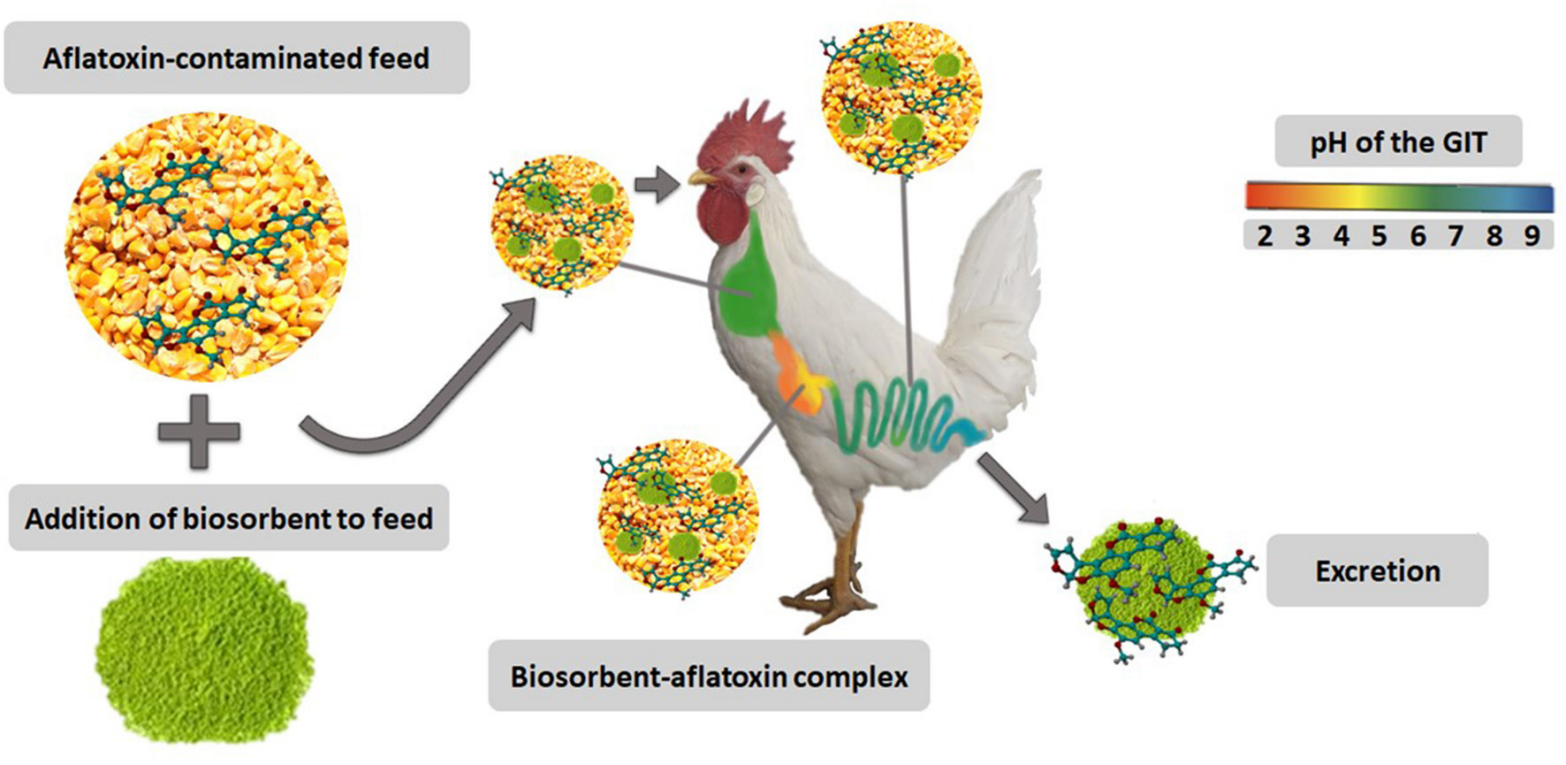
Biosorbents and their mode of action. Created with BioRender.com.

The increasing number of reports on the presence of aflatoxins in food and feedstuffs dictates the necessity of safe, practical, and economic decontamination procedures (7). Such procedures can be categorized into physical, chemical, and biological (Figure 2). In general, physical strategies are most effective than the other methods. Among these, the adsorption of aflatoxins onto various types of materials appears to be the most extensively used procedure. In this context, inorganic binders have been reported as the most efficient materials to remove aflatoxins *in vitro* and *in vivo* (8); however, facing the relative inefficacy of inorganic binders toward other mycotoxins, biosorption has been also suggested (9). Biosorption is a property of certain biomaterials to bind and concentrate selected ions or molecules from aqueous solutions (10). One of the major advantages of biosorption is its efficacy to remove aflatoxins—totally or up to satisfactory levels—(Figure 3) along with the recycling and/or usage of waste materials and byproducts. Biosorption technology for removing mycotoxins is not new; in 1980, Smith (11) reported that alfalfa and oat fibers significantly reduced the toxic effect of zearalenone on female weanling rats. Ever since that date, an increasing number of publications on the subject have appeared in the scientific literature. Many of these articles have tried to prove the *in vitro* and *in vivo* effectiveness of different biomaterials to adsorb mycotoxins such as yeast cell wall, lactic acid bacteria, activated carbon, and polymers. However, few of them have been reported the use of agro-waste-based materials for the adsorption of aflatoxins. In light of the growing interest in this rapidly evolving subject area, we will attempt to provide an update on the most important agro-waste-based materials used to adsorb aflatoxins. Further data of the *in vitro, in vivo*, and *in silico* experiments are also given. Moreover, a focus on the main characterization techniques used to elucidate the most important physical and chemical mechanisms involved in the adsorption process is presented. Finally, conclusions and future research necessities are also outlined.

## Types of Agro-Waste-Based Adsorbents

### Grape and Olive (Pomaces, Seeds, and Stems)

Grape (*Vitis vinifera* L.) is the largest fruit crop in the globe. In 2019, the grape production was over 98 million tons per year; about eighty percent was used in the winemaking industry, and the rest was for the preparation of juice, jams, and raisins (12). For this reason, the wine industry produces millions of tons of residues such as grape pomace (about 15 million tons worldwide). This plant-derived byproduct is inexpensive, available in large quantities, and is also known to contain significant amounts of valuable components that remain unexploited. Several processes have been suggested for its utilization; however, few of them have been focused on mycotoxin adsorption. The pomace (pulp and skins) obtained from Primitivo grape has been demonstrated to be an excellent aflatoxin adsorbent *in vitro* (13). In the research, the adsorption experiments were carried out at 37°C with 1 μg AFB_1_/mL. In general, the authors found that large particles yielded significantly lower adsorption uptakes; however, adsorption slightly increased by decreasing particle size (<500 μm). The maximum adsorption value recorded for AFB_1_ was 82% (Table 1). Moreover, the rate of aflatoxin adsorption was accomplished in a short period of time, 50% of adsorption occurred in 3 min, and the maximum was achieved in 15 min. Furthermore, the biomaterial was capable of adsorbing AFB_1_ to the same proportion in all tested pH values (from 3 to 9), and aflatoxin adsorption was significantly affected by the adsorbent dosage, the percentage of mycotoxins removed from neutral pH increased with increasing dosages of the biosorbent. By using the Langmuir model, the authors theoretically estimated the C_50_ (the adsorbent dosage to accomplish a 50% reduction of the toxin); thus, the C_50_ value for AFB_1_ was 1.2 mg/mL. Five years later, in another study from the same research group, Greco et al. (14) evaluated the ability of 51 agricultural byproducts (including fruit and grape pomaces) to adsorb AFB_1_. Biomaterials containing high levels of lignin, cellulose, and polyphenols were evaluated at a dosage of 10 mg/mL toward an aflatoxin working solution containing 1 μg/mL (Table 1). Significant AFB_1_-adsorption uptakes were reported for pomegranate byproducts (seeds and peels), artichoke, plantain peels, almond hulls, and carobs (up to 100% removal). In general, byproducts obtained from grapefruits (seeds and pomaces) adsorbed most aflatoxin. The mycotoxin-binding efficacy of grape pomaces is mainly related to the presence of micronized fibers and phenolic compounds. As the pH influences, the interaction between mycotoxin and biosorbent, Greco et al. (14) also conducted a desorption study to evaluate whether a change of pH can cause toxin release. The setup was as follows: AFB_1_ was adsorbed at pH 3, and then the aflatoxin-loaded biomaterials were washed with medium at pH 7. In general, grape pomaces and almond hull released <21% of the adsorbed AFB_1_. Additionally, Fernandes et al. (15) reported the *in vitro* adsorption of AFB_1_ by dry micronized olive pomace and grape stems. In the experiment, the biomaterials (20 mg/mL) were tested in buffer solutions containing aflatoxins (0.05, 0.5, 1, 2, 4, 6, and 10 μg/mL). Significant AFB_1_ adsorption efficiencies were observed (olive pomace 74% and grape stems 96%) in all tested pHs (2, 5, 7, and 8). In general, 30 mg/mL of olive pomace and 10 mg/mL of grape stems were necessary to achieve substantial adsorptions. Regarding desorption studies with buffer at pH 7, olive pomace was the biomaterial with the lowest capacity to retain the mycotoxin since desorption for AFB_1_ was as high as 40%. Grape stems retained the mycotoxin better; AFB_1_ was released only in a small amount, <5% (Table 1). Commonly, grape and olive pomaces, as well as grape stems, have good efficiencies in adsorbing mycotoxins at acid pH, and these biomaterials are also capable of retaining the adsorbed toxins when pH increases to 7. Consequently, the efficacy of two of these biomaterials (pomace from white and red grapes and almond hull) toward AFB_1_ was confirmed in an *in vivo* trial using urinary biomarkers as indicators of the absorbed mycotoxin in pigs (16). White grape pomace was the most effective biomaterial since it reduced 67% of the urinary biomarker AFM_1_ (Table 2). Recently, Taranu et al. (17) showed the efficacy of a grape seed waste in counteracting the toxic effects induced by AFB_1_ (320 ng/g) on productive parameters, plasma levels, liver damage, and intestinal tissues of pigs after weaning. The inclusion of grape seed in the aflatoxin-contaminated diet (8% w/w) enhanced the phase-I antioxidant enzymes activity, restored the pro-inflammatory cytokines and thiobarbituric acid reactive species (TBARS) levels, and improved the growth performance of the AFB_1_-intoxicated pigs (Table 2). These findings suggest that grape pomace and grape seed wastes are promising biomaterials in counteracting the harmful effects of AFB_1_ in pigs at higher inclusion levels (without adverse side effects). Unfortunately, the *in vivo* efficacies of olive pomace and grape stems have not been confirmed yet.

**Table 1:** *In vitro* effectiveness of different agro-waste-based materials to adsorb and desorb aflatoxins and the most important characteristic of the material related to the sorption.

**Biosorbent**	**Assay**	**Inclusion (%, w/w)**	**AFB_**1**_ (μg/mL)**	**Temperature (**°**C)**	**pH**	**Adsorption (%)**	**Desorption (%)**	**Characteristic related to the sorption**	**References**
Grape pomace (pulp and skins)	*In vitro*	0.5	1	37	3–8 range	82	4	NR	(13)
Almond hull	*In vitro*	1	1	37	7	87	6.4	High levels of lignin, cellulose, and polyphenols.	(14)
Carobs						100	NR		
Grape seeds						83	NR		
Grape pomace						94	Up to 8.6		
Pomegranate seeds						51	NR		
Pomegranate peel						55	NR		
Stalks and leaves of artichoke						55	21.2		
Plantain peel						67	NR		
Micronized grape stems	*In vitro*	2	1	37	2, 5, 7, and 8	96	< 5	NR	(15)
Micronized olive pomace						74	40		
Oven-dried banana peel	*In vitro*	6	0.5	22	3–9 range	Up to 74.9	< 13.6	Surface functional groups and the heterogeneous microstructure.	(18)
Unripe banana peel	*In vitro* (simulated poultry GIT conditions)	1.5	0.1	40	1.7, 5.2, and 6.7	28	NR	Surface functional groups and pigment content (chlorophylls, carotenoids, and anthocyanins).	(19)
*Pyracantha* leaves						46	NR		
*Aloe vera*						69	NR		
*Pyracantha* leaves	*In vitro*	0.5	0.1	40	4.8–5.4	86	NR	Surface functional groups, porosity and density (formation of agglomerates).	(20)
*Pyracantha* berries						46	NR		
Combination (leaves + berries)						82	NR		
Lignins from: *Rhododendron tomentosum*	*In vitro*	0.1	1	37	2	79.6	5.3	The total number of acidic hydroxyl groups and the capillary-porous structure.	(21)
*Althaea Officinalis*						80.2	1.3		
*Helianthus tuberosus*						71.7	14.3		
*Pícea*						50.4	12		
*Lavatera*						50.2	50.5		
Durian peel (*Durio zibthinus*)	*In vitro*/simulated GIT digestion process	0.5	1	37	3 and 7	Up to 46	NR	Porous structure, larger surface area, and higher surface charge.	(22)
Durian peel (acid-treated)						Up to 98.4	0		
Cellulose A	*In vitro*/simulating the digestion procedure of pigs	0.5	0.01	39	2 and 6.8	−31	NR	NR	(23)
Cellulose B						4	NR		
Lettuce (*Lactuca sativa* L.)	*In vitro*	0.5 and 0.1	0.19	40	2, 5, and 7	95	NR	Surface functional groups and the formation of AFB_1_-chlorophyll complexes.	(24)
Field horsetail (*Equisetum arvense* L.)						71	NR		
*Pyracantha* leaves						60	NR		
Lettuce (*Lactuca sativa* L.)	*In vitro*/Dynamic Gastrointestinal Tract-Simulated Model	0.5	0.1	40	2, 5 and 7	84	NR	Non-electrostatic interactions (hydrophobic interactions, dipole-dipole interactions, and hydrogen bonding) and electrostatic interactions (ionic attractions) together with the formation of AFB_1_-chlorophyll complexes.	(25)
Kale *(Brassica oleracea L.)*						94	NR		
*Lithothamnium calcareum*	*In vitro*	0.2	1	37	3 and 6	78	NR	NR	(26)

*GIT, gastrointestinal tract; NR, not reported*.

**Table 2:** *In vivo* efficacy of agro-waste-based materials in counteracting the harmful effects of aflatoxins.

**Biosorbent**	**Specie**	**Total animal number/(per experimental group)**	**Inclusion (%, w/w)**	**AFB_**1**_ (μg/g)**	**Efficacy**	**References**
White grape pomace	Weaned piglets	28/(4)	2.8	0.02	67% reduction for biomarker of AFB_1_ (AFM_1_).	(16)
Red grape pomace					None	
Pod pea					None	
Almond hull					None	
Grape seed meal	Weaned piglets	24/(6)	8	0.32	Ameliorated growth performance, decreased pro-inflammatory cytokines and TBARS levels, and enhanced the total capacity antioxidant in plasma and organs.	(17)
*Lithothamnium calcareum*	Male broiler chickens	64/(4)	0.2	1.018	Improved productive parameters, reduced the relative weight of the liver and macroscopic and microscopic changes, and improved some biochemical parameters.	(26)

*AFM_1_, aflatoxin M_1_; TBARS, thiobarbituric acid reactive species*.

### Banana Peel

Banana (*Musa sapientum* L.) is considered the world's fourth most important agricultural crop, with a worldwide production of about 102 million tons per year (12). It is then not surprising that banana peel —which constitutes about 30–40% of the total fruit weight— is a widely available byproduct (over 40 million tons per year worldwide). Several processes have been proposed for banana peel utilization; however, few of them have been focused on mycotoxin adsorption. In this context, Shar et al. (18) showed the effectiveness of banana peel for the *in vitro* elimination of aflatoxins (AFB_1_, AFB_2_, AFG_1_, and AFG_2_) at a concentration of 0.5 μg/mL of each toxin. In general, oven-dried banana peel was found to be more effective in removing aflatoxins. The optimum adsorbent dosage was 60 mg/mL, and most of the adsorption occurred in 10 min, while the maximum was reached in 30 min. The sorption capacity of banana peel increased with increasing pH (from 3 to 9). At pH 8, the maximum adsorption uptakes for AFB_1_, AFB_2_, AFG_1_, and AFG_2_ were 74.9, 63.1, 76.1, and 92.8%, respectively (Table 1). Desorption studies were also conducted, adsorptions were carried out at pH 3 and 8 and desorption at pH 8 and 3, respectively. In both cases, <10% of the adsorbed toxin was desorbed; thus, the adsorption of aflatoxins onto this biomaterial was strong enough to sustain pH changes. Recently, our research group showed—for the first time—the effectiveness of unripe banana peel in removing AFB_1_ using a laboratory setup simulating the *in vivo* environment of the poultry gastrointestinal tract (19). The gastrointestinal tract compartments simulated were the crop (pH 5.2), the proventriculus (pH 1.7), and the intestinal section (pH 6.7). A typical maize-soybean meal diet contaminated with 100 μg AFB_1_/kg was utilized, and the biosorbent was added into the diet at 1.5% w/w (Table 1). In general, when using this multi-compartmental model, banana peel presented a low AFB_1_-removal capacity (28%). This considerable variation in the efficiency of the biomaterial might be mainly due to the adsorbent dosage, the differences in banana species/cultivar, the maturity stage, the feed matrix effect, the consecutive incubation periods at different pHs, as well as the enzymatic activities utilized in the multi-compartmental model. In general, the efficacy of banana peel in removing aflatoxins is related to the presence of surface functional groups, the heterogeneous microstructure, and pigment content (chlorophylls, carotenoids, and anthocyanins). Further *in vitro* studies regarding toxin desorption and *in vivo* trials to evaluate the effectiveness of banana peel, however, need to be conducted.

### Formosa Firethorn (Leaves and Berries)

Formosa firethorn [*Pyracantha koidzumii* (Hayata) Rehder] is a spiny perennial shrub endemic in Taiwan. In many parts of the world, this one and some related species of the *Rosaceae* family are cultivated for decorative and walls purposes. Commonly, this plant is used in conventional medicine due to the diuretic, cardiac, and stimulant properties of its berries (27); however, limited evidence exists about other possible applications. In the scientific literature, some studies have been focused on the efficacy of *P. coccinea* berries for removing synthetic dyes (28–30). To date, there are only three studies —from our research group— regarding the biosorption potential of *P. koidzumii* against aflatoxins. Ramales-Valderrama et al. (20) reported the *in vitro* adsorption of AFB_1_ and AFB_2_ onto leaves, berries, and the combination of leaves and berries of *P. koidzumii*. The biosorbents were employed at 0.5% (w/v) in samples contaminated with 100 ng B-aflatoxins/mL. In the experiment, the ratio of AFB_1_ to AFB_2_ tested was 7:3, and adsorption was evaluated at 40°C up to 24 h. In general, the highest aflatoxin adsorption values were 86 and 82% using leaves and the combination of leaves and berries, respectively. A modest biosorption uptake (46%) was reported when using berries (Table 1). Unfortunately, most of the adsorption occurred in a long period of time (up to 6 h). Following this line, Zavala-Franco et al. (19), using a laboratory setup simulating the *in vivo* environment of the poultry gastrointestinal tract, showed the effectiveness of *Pyracantha* leaves in removing AFB_1_ (in this work, berries and the mixture of leaves/berries were not evaluated). All conditions used in the adsorption experiments were identical to those described in section Banana Peel. Using this multi-compartmental model, the authors indicated that the efficacy of the biomaterial was moderate; the biosorption uptake achieved was 46% (Table 1). However, neither desorption studies nor the *in vivo* effectiveness of these biosorbents have been reported yet. Very recently, using a theoretical perspective with density functional theory (DFT), Méndez-Albores et al. (31) showed the interaction of the AFB_1_ molecule and the chemical functional groups present in the surface of the *P. koidzumii* adsorbent. Hydroxyl, amino, carboxyl, and carbonyl groups were used as the characteristic functional groups on the biosorbent surface. *In silico* results showed that the carboxylate ion has the maximum binding energy with the AFB_1_ molecule (up to −40.2 kcal/mol); thus, the authors suggested that carboxylate ion-enriched adsorbents could be a very good option for AFB_1_ removal in *in vitro* and *in vivo* trials.

### Lignins and Micronized Fibers

Lignin is the main constituent of the xylem of almost all species of terrestrial plants. Several processes have been suggested for lignin application; however, few studies on the adsorption of mycotoxins have been reported. Until today, three *in vivo* studies demonstrate the effectiveness of dietary lignin in alleviating the adverse effects of deoxynivalenol and zearalenone in broiler chickens (32–34). Additionally, two *in vitro* studies, one regarding the structure and properties of lignin as an adsorbent for T-2 toxin (35) and the other concerning the adsorption-desorption of AFB_1_ on lignins (21), have been reported. In the context of this review, Karmanov et al. (21) showed the adsorption potential of lignins isolated from five medicinal plants (*Helianthus tuberosus, Atriplex patula, Rhododendron tomentosum, Althaea Officinalis*, and *Lavatera*) and lignins from the wood of spruce Pícea using an *in vitro* setup simulating the operational conditions of the digestion in the stomach of animals. In the experiment, the sorbents were utilized at 0.1% (w/v) in samples spiked with 1 μg AFB_1_/mL, which were incubated at 37°C for 30 min at pH 2. In general, lignins from *R. tomentosum* and *A. officinalis* exhibited the highest AFB_1_-adsorption capacities. In these samples, the AFB_1_-uptakes reached 79.6 and 80.2%, respectively (Table 1). Lignins from *Atriplex patula* presented the lowest ability to adsorb AFB_1_ (up to 41%). Furthermore, desorption studies conducted at pH 8 confirmed that lignins from *R. tomentosum* and *A. Officinalis* adsorbed irreversible the mycotoxin (<5.3% desorption). The selectivity of certain functional groups (OH) and the capillary-porous structure of lignins were responsible for their strong association with the AFB_1_ molecule. Although lignins have been proven *in vivo* to be effective in alleviating the adverse effects of deoxynivalenol, zearalenone, and T-2 toxin, there are no reports of their use in reducing the negative effects of aflatoxins using *in vivo* models. Furthermore, micronized fibers consisting mainly of cellulose, hemicellulose, and lignin, have also been reported as effective mycotoxin binders. It has been reported that micronized wheat fibers (up to 2% inclusion in diet) decrease the levels of ochratoxin-A in plasma, kidney, and liver of piglets and rats (36, 37). Very recently, Adunphatcharaphon et al. (22) showed the *in vitro* potential of the acid-treated durian (*Durio zibthinus*) peel for the adsorption of AFB_1_. Higher adsorption efficiency toward AFB_1_ (98.4%) was found for this biomaterial (Table 1). The acid-treated durian peel (mainly composed of 47.2% cellulose, 9.63% hemicellulose, and 9.89% lignin) was considered a promising byproduct for aflatoxin biosorption. In general, the porous structure, larger surface area, and higher surface charge were the principal physical characteristics of the acid-treated durian peel to remove aflatoxins. Nevertheless, other authors have suggested that the efficiency of cellulose products to adsorb AFB_1_ resulted not significant when proven in an *in vitro* model simulating the digestion of pigs (23) (Table 1).

### Aloe Vera

Aloe vera (*A. barbadensis* Miller) is a drought-resistant plant of the *Liliaceae* family. This plant has been used for medicinal purposes for over 5,000 years. Aloe vera gel is the mucilaginous extract of the leafy pulp, which is usually separated by scratching. It is well-known that the gel contains many phytochemicals (vitamins, minerals, enzymes, polysaccharides, and phenolic compounds) and has been claimed to have several curative and therapeutic properties (38). Although Aloe vera gel has been reported to exhibit several attractive properties (virucidal, bactericidal, and fungicidal) as well as the adsorbent ability against organic and inorganic pollutants, in the literature, there is only one report—from our research group—of its use as a biosorbent against AFB_1_ (19). In the work, the gel of matured leaves of *A. barbadensis* Miller was separated, pasteurized, and further concentrated to get a fine powder. The effectiveness of this powder in removing AFB_1_ was tested in a laboratory setup simulating the *in vivo* environment of the poultry gastrointestinal tract (all conditions used in the adsorption experiments were identical to those described in section Banana Peel). In general, Aloe vera powder limited the availability of AFB_1_ in the intestinal segment up to 69% (Table 1). The high negative-charged surface on the biosorbent was linked to the high sorption uptake due to enhancements of attractive forces between AFB_1_ and the biomaterial. However, further *in vivo* trials are needed to demonstrate its efficacy in reducing the toxic consequences of aflatoxins. Also, desorption studies will provide valuable information about the potential benefits of Aloe vera powder as a mycotoxin adsorbent biomaterial.

### Horticultural

Facing the relative inefficacy of some biosorbents toward mycotoxins, Nava-Ramírez et al. (24) investigated—for the first time—the *in vitro* potential of lettuce (*Lactuca sativa* L.) wastes and field horsetail (*Equisetum arvense* L.) in removing AFB_1_. The adsorption of AFB_1_ (190 ng/mL) was explored at two sorbent contents (0.5% and 0.1% w/v) and three pHs (3, 5, and 7). Adsorption was carried out at 40 °C for 2 h (Table 1). In general, at 0.5% (w/v), AFB_1_ was well-adsorbed by both biomaterials (70 to 100%). However, at 0.1% (w/v), lettuce showed the highest ability against AFB_1_ removal, the AFB_1_ biosorption percentage was 95% (at neutral pH). Adsorption was mainly due to the interaction of the AFB_1_ molecule with the functional groups of the biosorbents as well as to the formation of AFB_1_-chlorophyll complexes. Thus, the authors concluded that lettuce wastes could have significant potential for the removal of AFB_1_ in some gastrointestinal tract compartments at low inclusion levels. One year later, in another study by the same research group, Vázquez-Durán et al. (25) evaluated the adsorbent capacity of agricultural residues from kale (*Brassica oleracea* L.) using a dynamic *in vitro* model that simulated the conditions of the gastrointestinal tract of poultry. The biosorbent was added to a contaminated poultry feed (100 μg AFB_1_/kg) at a content of 0.5% (w/w). According to the adsorption results, the maximum adsorption capacity of kale was 93.6% in the intestinal section (Table 1). A biosorbent prepared from lettuce agro-waste was used as a reference, presenting a significantly lower adsorption percentage (83.7%). The researchers pointed out that adsorption of AFB_1_ may be mediated by simultaneous mechanisms, such as: non-electrostatic interactions (hydrophobic interactions, dipole-dipole interactions, and hydrogen bonds) and electrostatic interactions (ionic attractions). In addition, the authors also concluded that the formation of AFB_1_-chlorophyll complexes improved the rate of AFB_1_ adsorption. However, as recommended by Ramales-Valderrama et al. (20), biosorbents with high binding affinity for AFB_1_ *in vitro* need to be further tested *in vivo* to validate their efficacy to reduce the toxic effects of aflatoxins.

### Miscellaneous

Perali et al. (26) investigated the efficiency of the seaweed *Lithothamnium calcareum* (Pallas) Areschoug to remove AFB_1_. The study was conducted using two models, one *in vitro* and the other *in vivo*; the latter focused on evaluating the capacity of the adsorbent to prevent the toxic effects of AFB_1_ in broilers. The adsorption of AFB_1_ (1 μg AFB_1_/mL) was explored *in vitro* at four sorbent contents (0.05, 0.1, 0.15 and 0.2%) and two pHs (3 and 6). In general, highest percentages of AFB_1_ removal were achieved at a content of 0.2% in both evaluated pHs. In these samples, the AFB_1_-uptakes reached 77.6 and 77.4%, respectively (Table 1). Regarding the *in vivo* experiment, it was observed that the seaweed (0.2% inclusion) improved productive parameters (live weight, weight gain, and feed conversion ratio), reduced the relative weight of the liver and the macroscopic and microscopic changes caused by the AFB_1_ intoxication, and improved some biochemical parameters in birds that received a diet contaminated with 1,018 μg AFB_1_/g feed (Table 2).

## The Most Important Characterization Techniques for Biosorbent Characterization Before and After Aflatoxin Adsorption

### Fourier Transform Infrared (FTIR) Spectroscopy

FTIR spectroscopy is one of the most used techniques to characterize biosorbents, usually before and after mycotoxin adsorption. This methodology is relatively simple, reproducible, non-destructive, and only small quantities of biomaterials—without any further preparation—are required. Generally, FTIR spectroscopy provides information at the molecular level allowing investigation of functional groups, bonding types, and molecular conformations. In the FTIR spectra, most of the FTIR bands are relatively sharp and can be correlated with single bonds or particular functional groups. The position of a band is expressed into a plot with wavenumber (cm^−1^) on the *x*-axis and intensity on the *y*-axis. Intensity could be measured both in transmittance or absorbance modes. In the literature reviewed, most of the spectra were collected to recognize the main functional groups of the tested biosorbents. However, in the work of Ramales-Valderrama et al. (20), the FTIR spectra were acquired in order to elucidate the possible interaction mechanism between the AFB_1_ molecule and the biosorbents. In general, the authors indicated that a shift in the frequency (associated to an energy change) or a change in the band intensity confirm the involvement of a specific functional group in the aflatoxin binding. Table 3 summarizes the band assignments of the principal vibrational modes in the biomaterials used to remove aflatoxins. Biosorbents are mainly constituted of proteins, carbohydrates, lipids, and phytochemicals (curcuminoids, flavonoids, alkaloids, steroids, terpenoids, saponins, phenolics, glucosides, and chlorophylls). All of these components have several functional groups (hydroxyl, amino, carboxyl, carboxylate, amide, phosphate, ester, and ketone), which can be partially responsible for the biosorption of aflatoxins (Table 3). For instance, it has been reported that the hydroxyl, amino, carboxyl, and ester groups can efficiently establish hydrogen bonds with the oxygen atoms of the ether, carbonyl, and methoxy groups in the AFB_1_ molecule (25). Recently, theoretical infrared spectrophotometric studies of the adsorption of B-aflatoxins onto *Pyracantha* biosorbents showed that the carboxylate ion has the maximum binding energy with the AFB_1_ molecule. These *in silico* results imply—but do not yet prove—that an enriched biosorbent with carboxylate groups could increase the AFB_1_ adsorption (31). Finally, as an important remark, researchers might take into consideration that the main disadvantage of the FTIR technique is that several materials completely absorb IR radiation; therefore, it may be impossible to get reliable results.

**Table 3:** Overview of the most relevant chemical functional groups responsible for the biosorption of aflatoxins.

**Biomaterial**	**Aflatoxin removed**	**Wavenumber (cm^**−1**^)**	**Functional group**	**References**
Banana peel	AFB_1_, AFB_2_, AFG_1_, AFG_2_	3,500–3,200	OH stretching	(18)
		2,922	C–H stretching	
		1,734	C=O	
		1,600	COOR	
		1,380–1,300	C–H of the methyl, methylene, and methoxy	
		1,255–1,000	C–O stretching of carboxylic acids and alcohols	
*Pyracantha koidzumii* (leaves and berries)	AFB_1_, AFB_2_	3,360	OH and NH stretching	(20)
		1,738–1,638	C=O	
		1,070	PO_4_	
		832 and 765	–CH out of plane deformation in substituted aromatic hydrocarbons	
		630	C–CO–C bend in ketones	
Banana peel, *Pyracantha* leaves, and *Aloe vera* powder	AFB_1_	3,685–3,240	OH and NH stretching	(19)
		1,738–1,721	C=O stretching	
		1,091–1,073	(PO_2_) symmetric stretching	
		894–830	C–H out of plane deformation, NH_2_ wag	
		639–610	C–CO–C bend	
Durian peel	AFB_1_	3,300	OH stretching	(22)
		1,730	C=O stretching	
		1,622	(–CONH_2_)	
		1,500–1,200	Carboxylic, methyl, aromatic amines, and C–O stretching of carboxylic acids	
Lignin	AFB_1_	1,716	OH	(21)
		3,700–3,100	C=O in ester, aldehydes, and ketones	
Lettuce and field horsetail	AFB_1_	3,674–3,282	OH stretching	(24)
		1,733–1,608	C=O and COOR	
		1,315	C=O–N	
		1,242–1,027	PO_4_	
Lettuce and kale	AFB_1_	3,688–3,000	OH	(25)
		1,777–1,487	C=O and COOR	
		1,487–1,274	C=C	
		1,192–933	C-O	

### UV-Vis Diffuse Reflectance Spectroscopy (DRS)

The UV-Vis diffuse reflectance spectroscopy (DRS) is a useful technique for the characterization of biomaterials. Diffuse reflectance occurs as UV-Vis-light enters the sample, interacts with its components, and scatters backward. As a consequence, this technique provides information related to structural, physical, and chemical properties of the biomaterials and offers exceptional versatility because of its high sensitivity. Diffuse reflectance measurements make it possible to quickly and non-destructively evaluate—*in situ*—the content of chlorophylls, carotenoids, and anthocyanins in certain biomaterials. The most representative pigment in biosorbents is chlorophyll, of which the most common and abundant species are chlorophylls *a* and *b*. By wet chemical methods, a complex destructive procedure based on extraction and separation with organic solvents and spectrophotometric analysis is necessary for chlorophyll estimation. However, reflectance spectroscopy has been widely used for non-destructive estimation of chlorophylls in plant tissues. Limited information is available in the literature on the use of UV-Vis reflectance spectroscopy for pigment assessment in biosorbents used for aflatoxin removal. To date, there are only two reports using *Pyracantha* leaves, banana peel, Aloe vera, lettuce, and kale biosorbents (Table 1). Zavala-Franco et al. (19) and Vázquez-Durán et al. (25) found that almost all of the biosorbents presented the characteristic absorption bands at 677 and 650 nm, which correspond to chlorophyll *a* and chlorophyll *b*, respectively. Biosorbents also showed absorbance bands from 425 to 485 nm and at 550 nm indicative of the presence of carotenoids and anthocyanins, respectively. Furthermore, many studies have indicated that chlorophylls can form strong non-covalent complexes *in vitro* with AFB_1_ independent of temperature or pH. Consequently, the formation of a complex with the aflatoxins (via electrostatic, π-π orbital interactions, and/or hydrogen bonding) may be expected to improve the rate of AFB_1_ uptake by the biosorbents containing significant amounts of chlorophylls (24, 25).

### Zeta Potential (ζ) or Electrokinetic Potential

The adsorption of mycotoxins to a biomaterial surface in aqueous media could be based on a set of chemical and physical mechanisms, including hydrogen bonding, electrostatic attraction, ion exchange, chelation, precipitation, complexation, among others. Apparently, electrostatic interaction is the most important phenomenon during mycotoxin adsorption. Thus, zeta potential is important for the characterization of electrochemical surface properties since the electrokinetic potential at the electrical double layer is associated with the surface charge of colloidal suspensions. Both surface charge and environmental conditions—pH and ions in the medium—influence the zeta potential. Commonly, the zeta potential is determined by the micro-electrophoresis technique. In this procedure, a voltage is applied across a pair of opposite gold-plated electrodes; as a result, charged particles are attracted to the oppositely charged electrode and their velocity measured. The SI unit for electrophoretic mobility is μm cm/V s, since it is a velocity (μm/s) per field strength (V/cm). The electrophoretic mobility is the direct measurement from which zeta potential can be derived using the Helmholtz-Smoluchowski, Debye-Hückel, or Henry functions. Biosorbents with a zeta potential value between −10 and +10 mV are neutral, while those with zeta potentials >+30 mV or <-30 mV are strongly cationic or strongly anionic, respectively. In the literature, various research groups have reported the zeta potential of different biosorbents used for the removal of mycotoxins (19, 20, 22, 39). Table 4 summarizes the zeta potential values of the biomaterials used to adsorb aflatoxins. Considering that the interaction between aflatoxins and the biosorbent would be mainly electrostatic, biosorbents exhibiting higher zeta potential values are most adequate to be used in the adsorption due to the improvement of attractive forces between aflatoxin molecules and the surface of biomaterials. According to the reviewed literature, lettuce and field horsetail were the biosorbents with the high negative-charged surfaces (Table 4). However, up to now, none of the biosorbents shown in Table 4 have been tested *in vivo*.

**Table 4:** Zeta potential values of the agro-waste-based materials used for aflatoxins adsorption.

**Biomaterial**	**Zeta potential (– mV)**	**pH**	**Aflatoxin removed**	**References**
*Pyracantha koidzumii*:				
Leaves	21.8	4.8–5.4	AFB_1_ AFB_2_	(20)
Berries	17.2			
Leaves + Berries	23.2			
Banana peel	13.5	6.7	AFB_1_	(19)
*Pyracantha* leaves	28.0			
*Aloe vera* powder	17.5			
Durian peel	2.55	3	AFB_1_	(22)
Acid-treated durian peel	23.2			
Lettuce	30.0	7	AFB_1_	(24)
Field horsetail	40.0			
Lettuce	24	7	AFB_1_	(25)
Kale	18			

### Point of Zero Charge (pHzpc)

The point of zero charge (pHpzc) gives useful information about the surface charge of the biosorbents. It is well-known that pH influences sorption, mainly because pH governs the ionization of functional groups. In consequence, the pH at which the sorbent surface charge become equal to zero is defined as the point of zero charge. In other words, the charge of the positive surface sites is equal to that of the negative ones. It has been suggested that if pH < pHpzc, the surface of the biomaterial will be positively charged, and if pH > pHpzc, the surface will be negatively charged (40). Several methodologies have been reported for the determination of pHpzc, such as the potentiometric mass titration, the mass titration, and the immersion technique. In the literature, all of the biosorbents used for the adsorption of aflatoxins were characterized relative to its pHpzc by using the immersion technique. As an example, Akar et al. (39) reported pHpzc values of 1.9 and 2.7 for natural and modified sugar beet pulp wastes, respectively. The chemically modified biosorbent was used as an efficient material for zearalenone removal. Table 5 summarizes the pHzpc values of the biomaterials used for the *in vitro* adsorption of aflatoxins. Most of the biomaterials summarized in Table 5 have good efficiencies in adsorbing aflatoxins *in vitro*, but none of them have confirmed their effectiveness in *in vivo* trials. Considering the pH in the different compartments of the gastrointestinal tract of poultry, biosorbents with low pHpzc could be the most suitable to be used for the adsorption of aflatoxins when using *in vivo* models. Consequently, it would be interesting to study the *in vivo* effectiveness of the modified sugar beet pulp waste to remove aflatoxins since this biomaterial has the lowest pHpzc reported in the literature.

**Table 5:** Point of zero charge (pHpzc) values of the agro-waste-based materials used for aflatoxins adsorption.

**Biomaterial**	**pHpzc**	**Aflatoxin removed**	**References**
Banana peel (oven-dried)	5.5	AFB_1_, AFB_2_, AFG_1_, AFG_2_	(18)
Banana peel	6.7	AFB_1_	(19)
*Pyracantha* leaves	4.5		
*Aloe vera* powder	4.1		
Lettuce	5.7	AFB_1_	(24)
Field horsetail	5.7		
Lettuce	6.3	AFB_1_	(25)
Kale	6.2		

### Scanning Electron Microscopy (SEM) and Energy-Dispersive X-Ray Spectroscopy (EDS)

It is well-known that the adsorption properties of biomaterials may also be associated with both the structural and chemical features. In this context, the scanning electron microscopy (SEM) is one of the most versatile techniques available for the examination and analysis of the microstructure and morphology. Commonly, dried biosorbents must be mounted on special holders using a conductive carbon double-sided sticky tape. To increase image contrast and to avoid undesirable charging effects, it is necessary to coat the sample with a thin layer of a high electrical and thermal conductivity material such as gold, platinum, or carbon. Studies reporting the utilization of the SEM analysis for comparing the surface of biosorbents before and after aflatoxin adsorption are still meager (20). In the literature, most of the studies only report the morphological structure of the developed biosorbents previous aflatoxin exposure (18, 22). In general, more pores or cavities on the surface of the biomaterials provide higher capacities for aflatoxin adsorption. On the other hand, energy dispersive X-ray spectroscopy (EDS) is a chemical microanalysis technique typically performed in conjunction with SEM. In this technique, the atoms on the surface are excited by an electron stream, causing X-rays to be emitted. The energy of the X-ray is distinctive of the element from which the X-ray was produced. The chemical composition of certain biomaterials has been reported. Zavala-Franco et al. (19) evaluated the elemental composition of three biosorbents used for the removal of AFB_1_. The authors found that the main elements of banana peel, *Pyracantha* leaves, and *Aloe* powder were C and O, corresponding to 97.3, 99.2, and 85.7% of the total weight, respectively. They also observed other minor elements such as Na, Mg, Al, Si, P, S, Cl, K, and Ca. Adunphatcharaphon et al. (22) showed the elementary composition of the acid-treated durian peel employed as an aflatoxin binder. EDS analysis also revealed that C and O were the main elements that constitute the pristine biomaterial. However, the acidic treatment affected the elemental composition increasing the proportion of C, enhancing its AFB_1_ binding efficacy. Summarizing, surface characterization (morphology and microstructure) can be accomplished by SEM. When scanning electron microscope is accessorized with EDS, chemical microanalysis can also be conducted with 1–3% accuracy (41).

### X-Ray Diffraction (XRD)

X-ray diffraction (XRD) is another non-destructive characterization technique suitable to study phase, structure, orientation, and other structural features such as crystallite size, unit cell dimensions, crystallinity, and crystal defects. In this technique, diffraction patterns are formed by constructive interference of a monochromatic beam of X-rays scattered at different angles. In the analyzed samples, amorphous regions generate broad peaks, whereas crystalline regions produce sharp peaks. In general, X-ray diffractograms are collected using CuKα radiation (λ = 0.15406 nm) over the 2θ range (10 to 100 degrees) with a fixed power source. The XRD patterns of biosorbents are rarely presented in the literature due to the fact that most of the biomaterials used for mycotoxin adsorption are essentially carbonaceous. However, the XRD patterns for banana peel and *Pyracantha* leaves showed a distinctive amorphous structure based on broad diffraction peaks (19). Both biosorbents showed a strong diffraction peak at 20° (2θ) and few small diffraction peaks at around 5.6°, 14.8°, 17.3°, 22.8°, and 24.0°. These diffraction peaks were associated with the structure of semi-crystalline starch. Aloe powder also presented an amorphous phase and crystalline peaks for sylvite (KCl) and halite (NaCl). Moreover, the authors reported the degree of crystallinity of the three tested biosorbents. In general, the degree of crystallinity of banana peel, *Pyracantha* leaves, and Aloe powder differed significantly, yielding values of 19.1, 10.9, and 44.7%, respectively. In the sorption experiments, Aloe vera powder—the biomaterial with the highest crystallinity index—showed the maximum efficiency against AFB_1_ removal (68.5%). Although reduction in crystallinity leads to more reactive samples (42), the adsorption of aflatoxins depends on several characteristics of the biosorbents such as the functional group/type (amount), pigment content, surface charge, microstructure, morphology, elemental composition, degree of crystallinity, among others.

## Conclusions and Future Research Necessities

**Figure 4:**
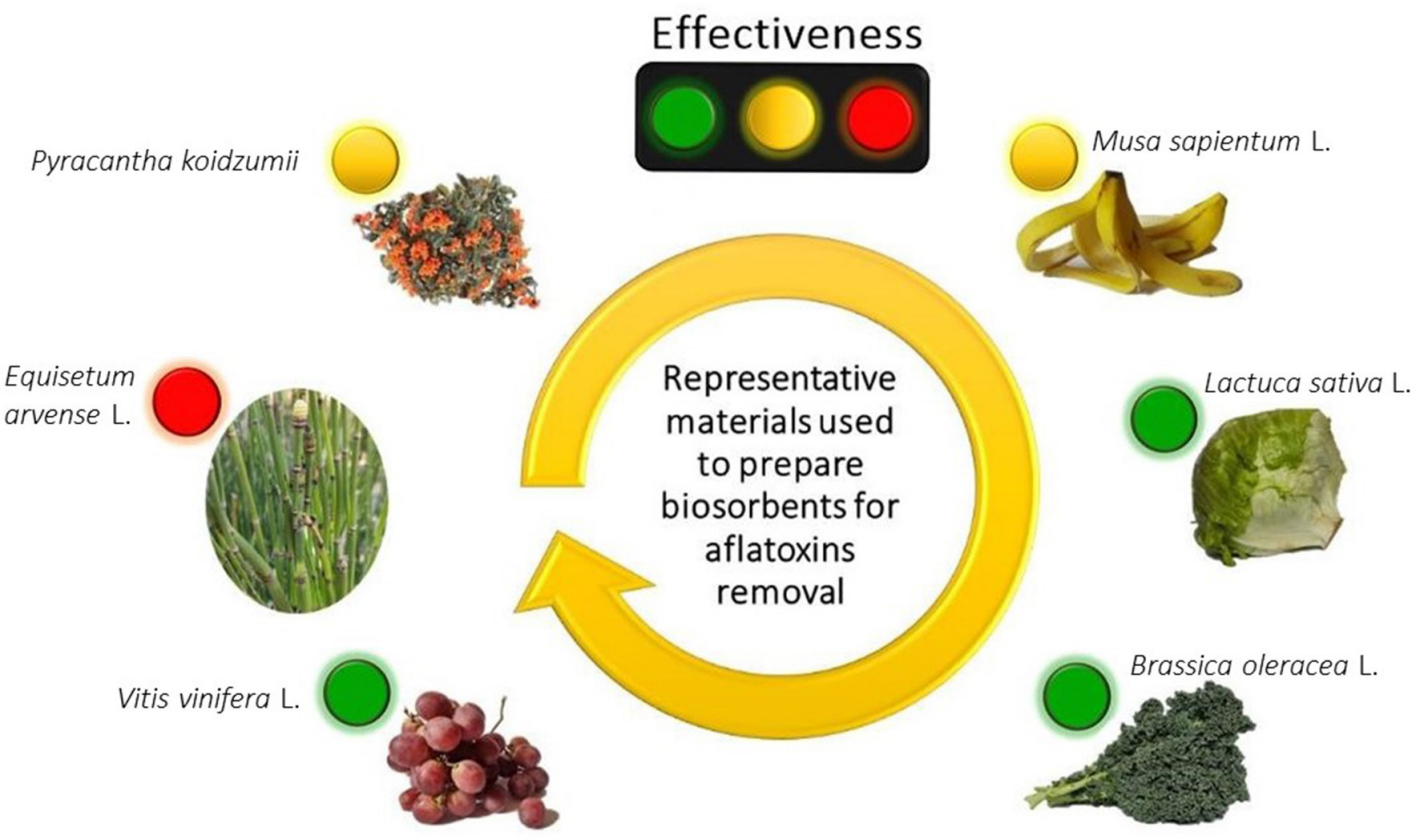
The effectiveness of some biosorbents to remove aflatoxins. Adsorption efficiency: red (low), yellow (moderate) and green (high). Created with BioRender.com.

Aflatoxins are inevitable contaminants of food and feed. Because of the adverse effects of aflatoxins on human and animal health, effective, practical, and inexpensive decontamination protocols are highly desirable. Recently, biosorption has received extensive attention among scientists for aflatoxin decontamination due to the low cost and the extraordinary efficiency of the biosorbents. Byproducts such as grape and olive (pomaces, seeds, and stems), banana peel, Formosa firethorn (leaves and berries), lignins, micronized fibers, durian peel, seaweeds, Aloe vera powder, lettuce, kale, and field horsetail have received particular attention for the removal of aflatoxins owing their abundance worldwide. As a result, several *in vitro, in vivo*, and *in silico* methodologies have been applied to evaluate the potential of these biosorbents in removing or reducing the impact of aflatoxins. A number of factors influencing the adsorption such as physical or chemical modification of the biomaterials, particle size, contact time, pH, temperature, biosorbent dosage, and the aflatoxin concentration, were further reviewed. We found the following:

- Structural changes following physical or chemical modifications of the biosorbents may explain their higher efficiencies in adsorbing aflatoxins.
- Biosorbents with large particles yielded lower adsorption uptakes. However, aflatoxin adsorption significantly increased by decreasing particle size.
- Generally, the rate of aflatoxin adsorption was accomplished in a short period of time (from 3 up to 30 min). This fast kinetic is highly desirable for practical and commercial applications.
- Various kinds of biosorbents have good efficiencies in adsorbing aflatoxins at acid pHs and were also capable of retaining most of the toxins when pH increases to 7, although some exceptions were observed.
- The biosorbents were efficient at temperatures between 37 and 40°C, which is indicative of their ability to adsorb aflatoxins when using *in vivo* trials.
- Further increments in the amount of biosorbents improve the uptake of aflatoxins due to the existence of more adsorption sites.
- Some biosorbents exhibited higher percentages of aflatoxin removal at lower toxin concentrations and considerable uptake capacities at higher aflatoxin concentrations (Figure 4).
- Generally, when using *in vitro* digestion procedures simulating the environment in the gastrointestinal tract, the tested biosorbents showed low removal efficiencies toward aflatoxins.
- In *in vivo* trials, some biosorbents counteracted the harmful effects of AFB_1_, but these were used at higher inclusion levels (up to 8% w/w).
- Several characterization techniques such as FTIR, UV-Vis DRS, ζ-potential, pHzpc, SEM, EDS, and XRD have been successfully used to explain the possible mechanisms involved in the biosorption of aflatoxins.

Despite the effectiveness of the biosorbents for the decontamination of aflatoxins, future research should be concentrated on the following topics:

- The effectiveness of biosorbents for removing aflatoxins needs to be extensively studied using dynamic models that simulate the conditions in the gastrointestinal tract on ways of minimizing the use of experimental animals.
- Biosorbents can be tested (*in vitro*) at low inclusion levels (0.1%, w/v) and challenged with more realistic levels of aflatoxins to make these materials very competitive in the commercial sorbent market.
- Taking into account that multi-exposure to mycotoxins is the most likely scenario, multi-mycotoxin adsorption experiments should be conducted in order to evaluate competitive biosorption.
- Because of the complex nature of the biosorbents, highly selective and sensitive analytical characterization techniques are necessary for a systematic characterization (before and after aflatoxin adsorption).
- Agro-waste-based sorbents such as cereal fibers as well as pulp and peels of some fruits may contain mycotoxins; consequently, these biomaterials need to be further analyzed before using them as mycotoxin binders.
- Novel approaches for the preparation of biosorbents from other agricultural wastes or byproducts are highly encouraged.
- Finally, in screening for new biosorbents, larger amounts of hydroxyl and carboxyl groups, high number of hydrophobic groups, higher amounts of pigments (chlorophylls), higher negative surface charge, lower pHpzc values, porous microstructure, and larger surface area seem to be the most important particularities to predict the ability of agro-waste-based materials to bind aflatoxins.

Considerations on these topics would help advance the search for near-term future commercial applications of unconventional, eco-friendly, and efficient aflatoxin binders of natural origin.

## Author Contributions

AV-D and AM-A: conceptualization. MN-R: investigation. AV-D: data curation. AM-A: writing—original draft preparation. AV-D and GT-I: writing—review and editing. All authors have read and agreed to the published version of the manuscript.

## Funding

This research was funded by UNAM-PAPIIT grant number IN207920.

## Conflict of Interest

The authors declare that the research was conducted in the absence of any commercial or financial relationships that could be construed as a potential conflict of interest.

## Publisher's Note

All claims expressed in this article are solely those of the authors and do not necessarily represent those of their affiliated organizations, or those of the publisher, the editors and the reviewers. Any product that may be evaluated in this article, or claim that may be made by its manufacturer, is not guaranteed or endorsed by the publisher.

## References

[1] Battilani P Gualla A Dall'Asta C Pellacani C Galaverna G Giorni P. Phomopsins: An overview of phytopathological and chemical aspects, toxicity, analysis and occurrence. World Mycotoxin J. (2011) 4:345–59. 10.3920/WMJ2011.1302

[2] Bhat R Rai RV Karim AA. Mycotoxins in food and feed: present status and future concerns. Compr Rev Food Sci F. (2010) 9:57–81. 10.1111/j.1541-4337.2009.00094.x 33467806

[3] Varga J Frisvad JC Samson R. Two new aflatoxin producing species, and an overview of Aspergillus section Flavi. Stud Mycol. (2011) 69:57–80. 10.3114/sim.2011.69.05 21892243 PMC3161756

[4] Frisvad JC Hubka V Ezekiel C Hong SB Nováková A Chen A. Taxonomy of Aspergillus section Flavi and their production of aflatoxins, ochratoxins and other mycotoxins. Stud Mycol. (2019) 93:1–63. 10.1016/j.simyco.2018.06.001 30108412 PMC6080641

[5] McLean M Dutton MF. Cellular interactions and metabolism of aflatoxin: an update. Pharmacol Ther. (1995) 65:163–92. 10.1016/0163-7258(94)00054-7 7540767

[6] Ostry V Malir F Toman J Grosse Y. Mycotoxins as human carcinogens—the IARC Monographs classification. Mycotoxin Res. (2017) 33:65–73. 10.1007/s12550-016-0265-7 27888487

[7] Mahato DK Lee KE Kamle M Devi S Dewangan K Kumar P. Aflatoxins in food and feed: an overview on prevalence, detection and control strategies. Front Microbiol. (2019) 10:2266. 10.3389/fmicb.2019.02266 31636616 PMC6787635

[8] Phillips TD Kubena LF Harvey RB Taylor DH Heidelbaugh ND. Hydrated sodium calcium aluminosilicate: a high affinity sorbent for aflatoxin. Poult Sci. (1988) 67:243–7. 10.3382/ps.0670243 2837754

[9] Jouany JP. Methods for preventing, decontaminating and minimizing the toxicity of mycotoxins in feeds. Animal Feed Sci Tech. (2007) 137:342–62. 10.1016/j.anifeedsci.2007.06.009

[10] Volesky B. Biosorption and me. Water Res. (2007) 41:4017–29. 10.1016/j.watres.2007.05.062 17632204

[11] Smith T. Influence of dietary fiber, protein and zeolite on zearalenone toxicosis in rats and swine. J Anim Sci. (1980) 50:278–85. 10.2527/jas1980.502278x 6244257

[12] Faostat F. Statistical Databases. Rome: Food and Agriculture Organization of the United Nations (2019).

[13] Avantaggiato G Greco D Damascelli A Solfrizzo M Visconti A. Assessment of multi-mycotoxin adsorption efficacy of grape pomace. J Agr Food Chem. (2014) 62:497–507. 10.1021/jf404179h 24364566

[14] Greco D D'Ascanio V Santovito E Logrieco AF Avantaggiato G. Comparative efficacy of agricultural by-products in sequestering mycotoxins. J Sci Food Agr. (2019) 99:1623–34. 10.1002/jsfa.9343 30187492

[15] Fernandes JM Calado T Guimarães A Rodrigues MAM Abrunhosa L. *In vitro* adsorption of aflatoxin B 1, ochratoxin A, and zearalenone by micronized grape stems and olive pomace in buffer solutions. Mycotoxin Res. (2019) 35:243–52. 10.1007/s12550-019-00349-9 30903558

[16] Gambacorta L Pinton P Avantaggiato G Oswald IP Solfrizzo M. Grape pomace, an agricultural by-product reducing mycotoxin absorption: *in vivo* assessment in pig using urinary biomarkers. J Agric Food Chem. (2016) 64:6762–71. 10.1021/acs.jafc.6b02146 27509142

[17] Taranu I Marin DE Palade M Pistol GC Chedea VS Gras MA. Assessment of the efficacy of a grape seed waste in counteracting the changes induced by aflatoxin B1 contaminated diet on performance, plasma, liver and intestinal tissues of pigs after weaning. Toxicon. (2019) 162:24–31. 10.1016/j.toxicon.2019.02.020 30849456

[18] Shar ZH Fletcher MT Sumbal GA Sherazi STH Giles C Bhanger MI. Banana peel: an effective biosorbent for aflatoxins. Food Addict Contam : Part A. (2016) 33:849–60. 10.1080/19440049.2016.1175155 27052947

[19] Zavala-Franco A Hernández-Patlán D Solís-Cruz B López-Arellano R Tellez-Isaias G Vázquez-Durán A. Assessing the aflatoxin B1 adsorption capacity between biosorbents using an *in vitro* multicompartmental model simulating the dynamic conditions in the gastrointestinal tract of poultry. Toxins. (2018) 10:484. 10.3390/toxins10110484 30469366 PMC6265716

[20] Ramales-Valderrama RA Vázquez-Durán A Méndez-Albores A. Biosorption of B-aflatoxins using biomasses obtained from formosa firethorn [*Pyracantha koidzumii* (Hayata) Rehder]. Toxins. (2016) 8:218. 10.3390/toxins8070218 27420096 PMC4963850

[21] Karmanov AP Kanarsky AV Kanarskaya ZA Kochevar LS Semenov EI Bogdanovich NI. *In vitro* adsorption-desorption of aflatoxin B1 on Pepper's lignins isolated from grassy plants. Int J Biol Macromol. (2020) 144:111–7. 10.1016/j.ijbiomac.2019.12.081 31838066

[22] Adunphatcharaphon S Petchkongkaew A Greco D D'Ascanio V Visessanguan W Avantaggiato G. The effectiveness of durian peel as a multi-mycotoxin adsorbent. Toxins. (2020) 12:108. 10.3390/toxins12020108 32046316 PMC7076778

[23] Kong C Shin SY Kim BG. Evaluation of mycotoxin sequestering agents for aflatoxin and deoxynivalenol: an *in vitro* approach. SpringerPlus. (2014) 3:346. 10.1186/2193-1801-3-346 25045616 PMC4101124

[24] Nava-Ramírez MJ Salazar AM Sordo M López-Coello C Téllez-Isaías G Méndez-Albores A. Ability of low contents of biosorbents to bind the food carcinogen aflatoxin B1 *in vitro*. Food Chem. (2021) 345:128863. 10.1016/j.foodchem.2020.128863 33340893

[25] Vázquez-Durán A Nava-Ramírez MJ Hernández-Patlán D Solís-Cruz B Hernández-Gómez V Téllez-Isaías G. Potential of kale and lettuce residues as natural adsorbents of the carcinogen aflatoxin B1 in a dynamic gastrointestinal tract-simulated model. Toxins. (2021) 13:771. 10.3390/toxins13110771 34822555 PMC8617829

[26] Perali C Magnoli AP Aronovich M Rosa CADR Cavaglieri LR. *Lithothamnium calcareum* (Pallas) Areschoug seaweed adsorbs aflatoxin B1 *in vitro* and improves broiler chicken's performance. Mycotoxin Res. (2020) 36:371–9. 10.1007/s12550-020-00402-y 32666398

[27] Kowalewski Z Mrugasiewicz K. Neue flavanonheteroside in *Crataegus phenophyrum*. Planta Med. (1971) 19:311–7. 10.1055/s-0028-1099647 5103297

[28] Akar T Anilan B Gorgulu A Akar ST. Assessment of cationic dye biosorption characteristics of untreated and non-conventional biomass: *Pyracantha coccinea* berries. J Hazard Mater. (2009) 168:1302–9. 10.1016/j.jhazmat.2009.03.011 19362415

[29] Akar T Celik S Akar ST. Biosorption performance of surface-modified biomass obtained from *Pyracantha coccinea* for the decolorization of dye contaminated solutions. Chem Eng J. (2010) 160:466–72. 10.1016/j.cej.2010.03.047

[30] Ari AG Celik S. Biosorption potential of Orange G dye by modified *Pyracantha coccinea*: batch and dynamic flow system applications. Chem Eng J. (2013) 226:263–70. 10.1016/j.cej.2013.04.073

[31] Méndez-Albores A Escobedo-González R Aceves-Hernández JM García-Casillas P Nicolás-Vázquez MI Miranda-Ruvalcaba R. Theoretical study of the adsorption process of B-aflatoxins using *Pyracantha koidzumii* (Hayata) Rehder biomasses. Toxins. (2020) 12:283. 10.3390/toxins12050283 32354011 PMC7290487

[32] Grešáková L Borutová R Faix Š Plachá I Cobanová K Košíková B. Effect of lignin on oxidative stress in chickens fed a diet contaminated with zearalenone. Acta Vet Hung. (2012) 60:103–14. 10.1556/avet.2012.009 22366136

[33] Klapáčová K Faixová Z Faix Š Miklósová L Leng L. Effects of feeding wheat naturally contaminated with Fusarium mycotoxins on blood biochemistry and the effectiveness of dietary lignin treatment to alleviate mycotoxin adverse effects in broiler chickens. Acta Vet. (2011) 61:227–37. 10.2298/AVB1103227K

[34] Revajová V Levkut M Levkutová M Borutová R Grešaková L Košiková B. Effect of lignin supplementation of a diet contaminated with Fusarium mycotoxins on blood and intestinal lymphocyte subpopulations in chickens. Acta Vet Hung. (2013) 61:354–65. 10.1556/avet.2013.023 23921347

[35] Kanarskaya Z Kanarskii A Semenov E Karmanov A Kochevar L Bogdanovich N. Structure and properties of lignin as an adsorbent for mycotoxin T-2. Chem Nat Compd. (2016) 52:1073–7. 10.1007/s10600-016-1864-4

[36] Aoudia N Call P Grosjean F Larondelle Y. Effectiveness of mycotoxin sequestration activity of micronized wheat fibres on distribution of ochratoxin A in plasma, liver and kidney of piglets fed a naturally contaminated diet. Food Chem Toxicol. (2009) 47:1485–9. 10.1016/j.fct.2009.03.033 19345713

[37] Aoudia N Tangni E Larondelle Y. Distribution of ochratoxin A in plasma and tissues of rats fed a naturally contaminated diet amended with micronized wheat fibres: effectiveness of mycotoxin sequestering activity. Food Chem Toxicol. (2008) 46:871–8. 10.1016/j.fct.2007.10.029 18068288

[38] Langmead L Makins R Rampton D. Anti-inflammatory effects of aloe vera gel in human colorectal mucosa *in vitro*. Aliment PharmTher. (2004) 19:521–7. 10.1111/j.1365-2036.2004.01874.x 14987320

[39] Akar T Güray T Yilmazer DT Tunali Akar S. Biosorptive detoxification of zearalenone biotoxin by surface-modified renewable biomass: process dynamics and application. J Sci Food Agric. (2019) 99:1850–61. 10.1002/jsfa.9379 30264397

[40] Lim LB Priyantha N Tennakoon D Chieng HI Dahri MK Suklueng M. Breadnut peel as a highly effective low-cost biosorbent for methylene blue: equilibrium, thermodynamic and kinetic studies. Arab J Chem. (2017) 10:S3216–28. 10.1016/j.arabjc.2013.12.018

[41] Karimi K Taherzadeh MJ. A critical review of analytical methods in pretreatment of lignocelluloses: composition, imaging, and crystallinity. Bioresour Technol. (2016) 200:1008–18. 10.1016/j.biortech.2015.11.022 26614225

[42] Lopičić ZR Stojanović MD Marković SB Milojković JV Mihajlović ML Radoičić TSK. Effects of different mechanical treatments on structural changes of lignocellulosic waste biomass and subsequent Cu (II) removal kinetics. Arab J Chem. (2019) 12:4091–103. 10.1016/j.arabjc.2016.04.005

